# A box-shaped shielding device for reducing the risk of COVID-19 droplet infection during gastrointestinal endoscopic procedures

**Published:** 2020-12-11

**Authors:** Hideaki Kawabata, Yuji Okazaki, Kazuhisa Watanabe, Takato Inoue, Katsutoshi Yamaguchi, Yuki Ueda, Misuzu Hitomi, Masatoshi Miyata, Shigehiro Motoi

**Affiliations:** Department of Gastroenterology, Kyoto Okamoto Memorial Hospital, Kumiyama-cho, Kyoto, Japan

**Keywords:** shielding box, COVID-19, gastrointestinal endoscopy, endoscopic retrograde, cholangiopancreatography, infection prevention

## Abstract

**Background and Aims:**

Endoscopists and endoscopic assistants are easily exposed to germs, including COVID-19, during aerosol-generating procedures such as gastrointestinal endoscopy. This retrospective study investigated the utility of a box-shaped shielding device for reducing the risk of COVID-19 droplet infection during endoscopic procedures.

**Methods:**

We created a cuboid box (500 × 650 × 450 mm) with four sides were covered with a transparent, vinyl-chloride sheet having two windows for endoscopic passage and assistance. The shielding box was then placed over a patient’s head and shoulders and covered with another transparent vinyl sheet. We assessed its utility and safety using the medical data concerning the procedure time and vital signs and a questionnaire for the endoscopic staff and patients.

**Results:**

We performed endoscopic retrograde cholangiopancreatography-related procedures using this device for two patients suspected of having COVID-19-associated pneumonia. Both patients were smoothly and successfully treated without any complications. No difficulties were noted with either endoscopic operation or in assisting the procedure, and the transparency was good enough to observe the patients’ faces and movements.

**Conclusions:**

This box-shaped shielding device can be used to reduce the risk of COVID-19 droplet infection during endoscopic procedures in the clinical setting.

**Relevance for patients:**

The COVID-19 outbreak has reminded healthcare personnel working in endoscopy units of the importance of infection prevention during endoscopy. The box-shaped shielding device can help endoscopic staff avoid hospital-setting COVID-19 infection.

## 1. Introduction

The coronavirus disease 2019 (COVID-19) outbreak has reminded healthcare personnel working in endoscopy units of the importance of infection prevention during endoscopy [1-3]. Human-to-human transmission occurs primarily through direct contact or air droplets [4]. The greatest risk of transmission is within approximately 1 m from the infected person [5]. Endoscopists and endoscopic assistants are easily exposed to such germ not only by directly touching endoscopes that are contaminated with patients’ respiratory secretions but also by close proximity exposure to unexpected contaminated droplets produced by patients’ coughing, belching, or vomiting.

Several countermeasures have been recommended to prevent contamination, including narrowing endoscopic indications, wearing personal protective equipment (PPE), and operating in negative-pressure rooms [1-3,6]. However, emergent endoscopic procedures for patients with acute gastrointestinal (GI) bleeding, foreign bodies in the upper GI tract, obstructive jaundice, or acute ascending cholangitis should always be performed [2], and such urgent situations may distract medical staff from taking care to avoid personal contamination. Furthermore, PPE shortages and the lack of widespread access to negative-pressure endoscopy rooms [3,7] can also increase the risk of viral transmission. We should also remember that molecular tests that detect viral RNA can produce false-negative results [8].

The “aerosol box” is a novel device that shields doctors from droplet infection of coronavirus while intubating patients [9], and such devices have already been used in clinical practice [10,11]; however, we have found no similar devices suitable for use during endoscopy.

Therefore, we developed a box-shaped shielding device to reduce the risk of COVID-19 droplet infection during endoscopic procedures and herein report its utility in the clinical setting.

## 2. Patients and Methods

We retrospectively reviewed the clinical data of all consecutive patients who were suspected of having COVID-19-associated pneumonia and underwent endoscopic procedures with a shielding device in our institution between April 2020 and May 2020.

**Figure 1:**
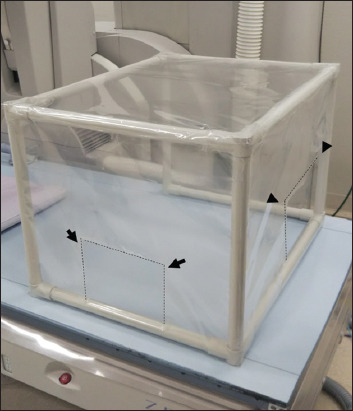
Four of six sides of a plastic, cuboid framework (500 × 650 × 450 mm) were covered with a transparent, vinyl-chloride sheet, which had two windows: One (150 × 180 mm) on the front side for passing an endoscope through (arrows) and another (200 × 400 mm) at the head side for assistance maneuvers (arrowheads).

The shielding device is shown in Figure 1. It consists of a plastic, cuboid framework (500 × 650 × 450 mm), with four sides covered with a transparent, vinyl-chloride sheet. This shielding box has two windows: One (150 × 180 mm) on the front side for passing an endoscope through and another (200 × 400 mm) at the head side for assistance maneuvers, such as suction.

**Figure 2:**
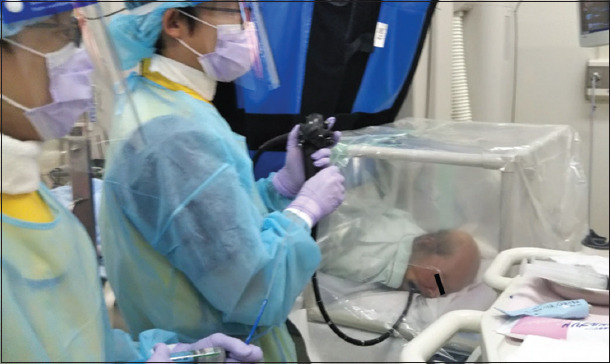
The shielding box was placed over the patient’s head and shoulders. In addition, the box was covered with another transparent vinyl sheet, which shielded the windows and stuck to the box using adhesive tape.

Before starting endoscopic procedures, patients were asked to place themselves into the prone position or left lateral position, and 2 L/min of oxygen was administered via nasal cannula. The heart rate, SpO_2_, respiratory rate, electrocardiogram findings, and non-invasive blood pressure were monitored during the procedure. Sedative drugs (midazolam and pentazocine) and antispasmodic drug (glucagon) were then administered intravenously. After confirming the sedative condition, the shielding box was placed over the patient’s head and shoulders. In addition, the box was covered with another transparent vinyl sheet that shielded the windows and was stuck to the box using adhesive tape (Figure 2). After the procedure, the vinyl sheet was discarded, while the shielding box was disinfected with alcohol and reused.

We extracted the medical data concerning the time required for endoscope insertion and the endoscopic procedure as well as vital signs before, during, and after the procedure. We then asked the operators and assistant nurses to answer a questionnaire to assess the operability, visibility (transparency), and sense of security against droplet exposure using a numerical rating scale (1, poor; 5, excellent). In addition, we interviewed the patients about their impressions during the procedure.

All of the patients gave their written informed consent. This study followed the ethical guidelines for studies involving human subjects based on the Helsinki declaration. The study protocol was approved by the institutional review board of the Kyoto Okamoto Memorial Hospital.

## 3. Results

We performed endoscopic retrograde cholangiopancreatography (ERCP)-related procedures using this shielding device for two male patients in their 80s. The total amount of sedative drugs, procedure duration, and vital signs before, during, and after the procedure of each patient are shown in Table 1. One patient (Case 1) suffered from acute cholangitis due to choledocholithiasis and was suspected of having COVID-19-associated pneumonia based on computed tomography (CT) findings. ERCP had to be started before the polymerase chain reaction (PCR) results for a COVID-19 test were obtained due to severe inflammation with septic shock that required the administration of norepinephrine. He underwent endoscopic sphincterotomy (EST) followed by biliary stenting. The total procedure time was 12 min, and it took 2 min to achieve endoscope insertion. Instability of vital signs, except for the SpO_2_ level, was observed due to his septic shock; however, he was smoothly and successfully treated without any complications.

**Table 1:** Total amount of sedative drugs, the procedure duration, and vital signs before, during, and after the procedure in each patient.

Case	Sedative drugs (total, mg)		Total procedure time (min)	Endoscope insertion time (min)	Blood pressure (mmHg)			Heart rate (/min)			SpO_2_ (%)			Respiratory rate (min)		
				
	Midazolam	Pentazocine			Before	During	After	Before	During	After	Before	During	After	Before	During	After
1	2.5	7.5	12	2	97/62	76–96/52–60	118/70	96	93–98	90	97	97–98	98	18	19–22	25
2	3	7.5	10	3	102/65	99–101/64	93/62	91	79–81	82	97	96–97	96	15	15–17	15

Another patient (Case 2) who had a history of EST for choledocholithiasis suffered from an attack due to recurrent choledocholithiasis. He was suspected of having COVID-19-associated pneumonia based on CT findings, although PCR for COVID-19 was ultimately negative. He underwent endoscopic stone removal. The total procedure time was 10 min, and it took 3 min to achieve endoscope insertion. All vital signs were stable during the procedure, and he was also smoothly and successfully treated without any complications.

The assessment of the shielding device by an operator and an assistant nurse in each case is shown in Table 2. The rating scores obtained from the operator and assistant nurse were the same, with the operability and visibility (transparency) being rated 4/5 and the sense of security being rated 5/5; the scores were the same in both cases. Neither patient recalled the endoscopic procedure.

**Table 2:** The assessment of the shielding device by an operator and an assistant nurse in each case using a numerical rating scale (1, poor; 5, excellent).

Case	Operator			Assistant nurse		
	
	Operability	Visibility	Sense of security	Operability	Visibility	Sense of security
1	4	4	5	4	4	5
2	4	4	5	4	4	5

## 4. Discussion

In the present study, we mainly assessed three concerns associated with using a shielding device during endoscopic procedures: Operability, visibility (transparency), and risk of hypoxia due to shielding a patient. We also demonstrated its utility and safety in the clinical setting.

First, no difficulties were noted using this shielding device for GI endoscopy with either the endoscopic operation itself or in assisting the procedure, including with regard to the secretory suction and head fixation. Begley [12] reported aerosol boxes for intubation may increase intubation times and therefore expose patients to the risk of hypoxia. However, it did not take long for the procedure to be performed, including endoscopic insertion, and the assessment of operability by the operators was satisfactorily high.

Second, the transparency of this device was good enough to observe the patients’ faces and movement despite having to see through two vinyl sheets while using this device, findings that differed from those with an aerosol box made of acrylic or transparent polycarbonate sheet. Fogging inside the device was also not observed during the procedure, although the procedure time was not very long.

Third, no deterioration of SpO_2_ was observed during the procedure. The risk of hypoxia due to shielding a patient was able to be avoided as not only was nasal O_2_ administrated, but the foot end of the box was open enough for ventilation. By adjusting the position of the box windows, this device can be used even for patients with severe respiratory conditions such as those under intubation, provided their general condition is deemed sufficient to tolerate endoscopic procedures.

Furthermore, it can provide endoscopic staff a psychological sense of security against droplet exposure. However, endoscopy under sedation is recommended to relieve compressive or occlusive feelings induced by the shielding device.

The box affected neither the position of endoscopy personnel nor the positioning of the C-Arm or the fluoroscopy during ERCP. The size of the box can be adjusted to the size of the fluoroscopic table and C-Arm. In addition, this device can also be used during other endoscopic procedures, including colonoscopy, bronchoscopy and laryngoscopy, provided the position of the box windows is modified to allow such endoscopes to be easily maneuvered.

However, it seems to be difficult to replace the use of PPE with this device, as protection is still required before and after the procedure and the device may fail to protect from aerosols (the small respirable particles <5–10 µm in diameter) that can remain airborne and are capable of short- and long-range transport [13]. Therefore, the box is expected to be used as an additional protection device and is expected to be particularly useful in case of otherwise insufficient infection protection, such as under condition of PPE shortage and emergent situation. In addition, the vinyl sheet around the framework should be discarded completely after each usage, as the reuse of the box within a short span of time even after alcohol disinfection may induce cross contamination between patients.

Larger, prospective, multicenter studies including GI endoscopic procedures, such as hemostasis, are needed to clarify the utility and safety of this device. The feasibility of the device’s practical application to other endoscopic procedures including colonoscopy, bronchoscopy and laryngoscopy, after the position of the box windows are adjusted should also be assessed.

Furthermore, experimental studies concerning the reduction in aerosol spray when using the box should be conducted. For example, a study measuring the concentration of aerosols in the box and at several points in the endoscopy room before, during and after the procedure, with the results compared to those obtained without the box should be conducted.

In conclusion, this box-shaped, shielding device can be used to reduce the risk of COVID-19 droplet infection during endoscopic procedures in the clinical setting. We hope that this device will help endoscopic staff to avoid hospital-setting COVID-19 infection.

### Conflicts of Interest

The authors declare that they have no conflicts of interest.
